# Rising Kratom Use and Toxicity: An Observational Study, 2015-2024

**DOI:** 10.1192/j.eurpsy.2026.10481

**Published:** 2026-07-31

**Authors:** E. B. Towers, Y. T. Thomas, C. P. Holstege, R. Farah

**Affiliations:** 1 Medical Scientist Training Program, University of Virginia, Charlottesville; 2 Henry J.N. Taub Department of Emergency Medicine, Baylor College of Medicine, Houston; 3 Department of Emergency Medicine, Division of Medical Toxicology, University of Virginia, Charlottesville, United States

## Abstract

**Introduction:**

Kratom (*Mitragyna speciosa*), a botanical native to Southeast Asia, was traditionally consumed for pain relief and opioid withdrawal. Today, it is marketed as a “natural wellness” product and sold in gas stations, vape shops, and online with minimal oversight. Its shift from whole leaf to high-potency formulations, still labeled as kratom but often enriched with 7-hydroxymitragynine, has raised growing concerns about toxicity.

**Objectives:**

To determine recent changes in kratom use and medically attended kratom-related exposures in the United States (U.S.).

**Methods:**

A retrospective analysis of kratom use and kratom-related exposures was conducted using data from the U.S. National Survey on Drug Use and Health (NSDUH; 2019–2023) and the U.S. National Poison Data System (NPDS; 2015–2024). Analyses included individuals aged 12 years and older in the U.S. and assessed trends in demographics, clinical outcomes, and substances co-used with kratom.

**Results:**

From 2019–2023, NSDUH estimated lifetime kratom use rose from 4.0 to 5.1 million, while past-year (1.6–2.1 million) and past-month use (0.83–1.14 million) remained stable (Figure 1). Males comprised 50–67% of users. NPDS recorded 11,015 medically attended exposures during 2015–2024, increasing from 258 to 1,524 (Figure 2A); 35–43% involved co-use. Most exposures occurred in males (66–76%) and adults aged 20–39, but exposures are rising among older adults (40–59 and >60) and adolescents (Figure 2B). Compared with kratom-only exposures, co-use exposures had nearly double the hospitalization rate (44–56% vs. 24–28%) and more frequent critical care admissions (20–31% vs. 9–12%; Figure 3A). Serious outcomes, including death, were also more common with co-use (56–65%) than kratom alone (42–49%; Figure 3B). Deaths totaled 204, including 169 with co-use. Common co-used substances (ethanol, opioids, benzodiazepines, stimulants, and antidepressants) were also most frequent in hospitalizations and serious outcomes, with opioids predominating in fatalities.

**Image 1:**
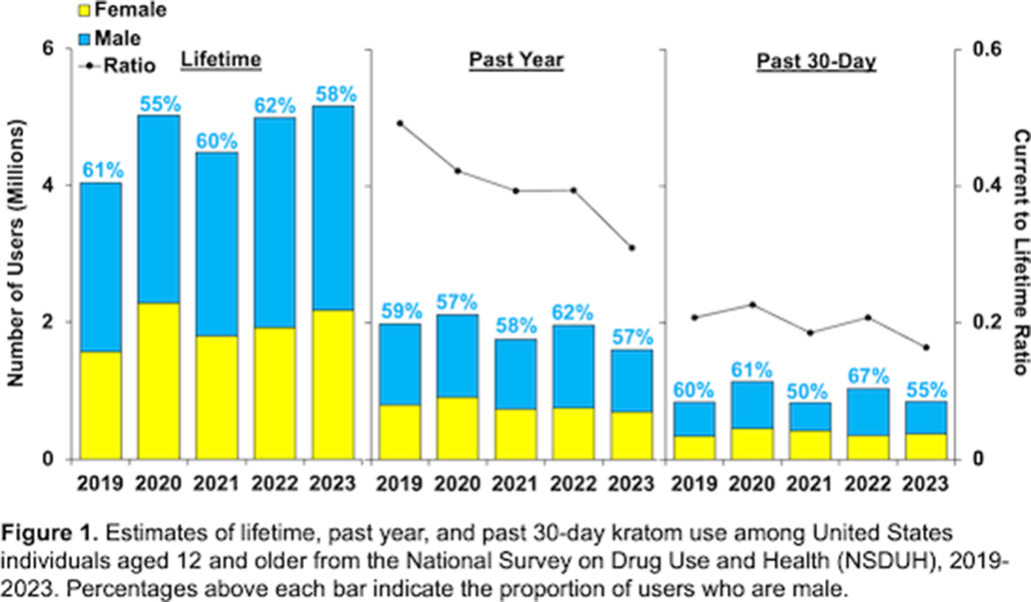
Image 1: Long description.

**Image 2:**
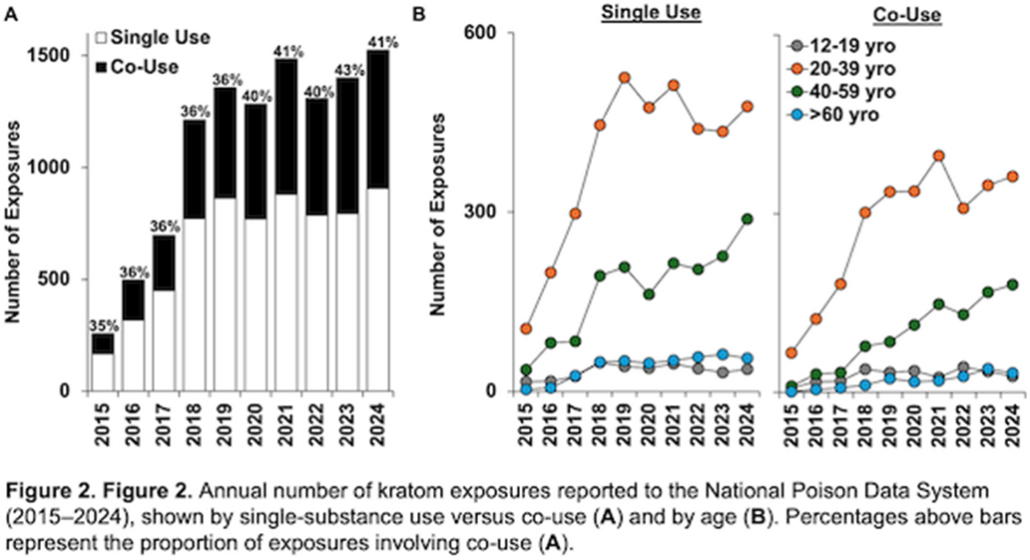
Image 2: Long description.

**Image 3:**
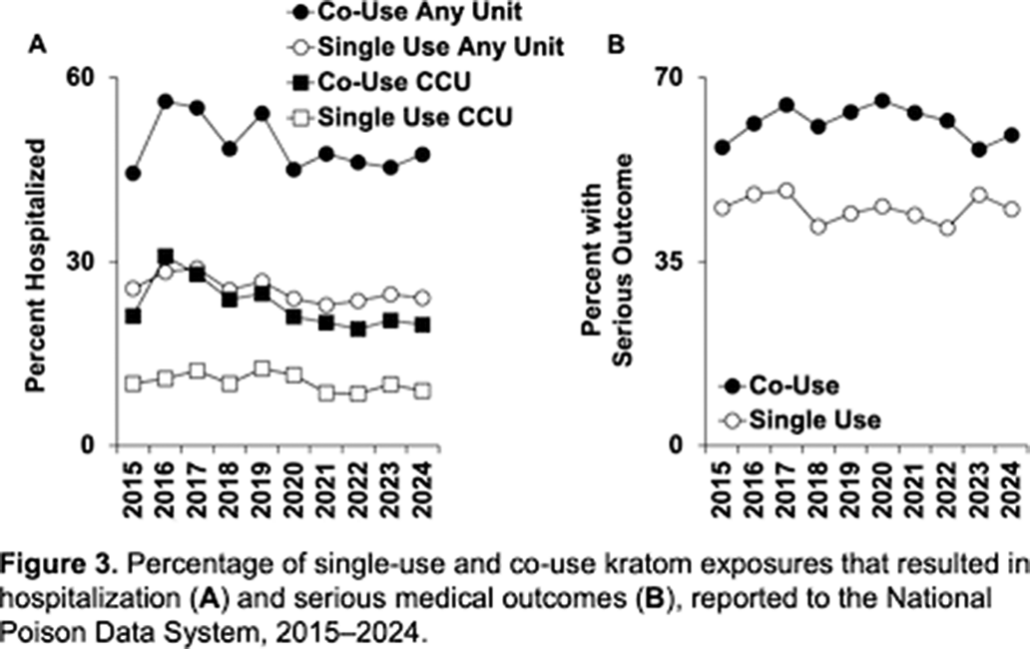
Image 3: Long description.

**Conclusions:**

Kratom use in the U.S. is rising, with medically attended exposures peaking in 2024 alongside the introduction of high-potency formulations enriched with 7-hydroxymitragynine. Hospitalizations and severe outcomes are largely tied to polysubstance use involving psychoactive drugs. These findings highlight the urgent need for enhanced surveillance, regulation of high-potency products, and targeted education to reduce harm in a rapidly evolving drug landscape.

**Disclosure of Interest:**

None Declared

## Long descriptions

### Image 1: Long description

The graph uses two y-axes. The left axis measures the Number of Users in millions from 0 to 6. The right axis measures the Current to Lifetime Ratio from 0 to 0.6. The x-axis for all panels shows years from 2019 to 2023. Stacked bars represent total users, with yellow for Female and blue for Male. A black line with circular markers represents the Ratio.

* Lifetime Panel: Total users fluctuate between 4 and 5 million. Male percentages are 61 percent in 2019, 55 percent in 2020, 60 percent in 2021, 62 percent in 2022, and 58 percent in 2023.

* Past Year Panel: Total users are lower, around 2 million. Male percentages are 59 percent in 2019, 57 percent in 2020, 58 percent in 2021, 62 percent in 2022, and 57 percent in 2023. The ratio line shows a downward trend from approximately 0.5 to 0.3.

* Past 30-Day Panel: Total users are the lowest, around 1 million. Male percentages are 60 percent in 2019, 61 percent in 2020, 50 percent in 2021, 67 percent in 2022, and 55 percent in 2023. The ratio line fluctuates between 0.15 and 0.25.

Navigate back to Image 1:.

### Image 2: Long description

Panel A is a stacked bar chart titled Number of Exposures. The X-axis shows years from 2015 to 2024. The Y-axis ranges from 0 to 1500. Each bar is divided into white (Single Use) and black (Co-Use). Total exposures increase from approximately 250 in 2015 to over 1500 in 2024. Percentages for Co-Use are labeled above each bar: 35% in 2015, 36% from 2016 to 2018, 36% in 2019, 40% in 2020, 41% in 2021, 40% in 2022, 43% in 2023, and 41% in 2024.

Panel B contains two line graphs sharing a Y-axis from 0 to 600.

1. The first graph, Single Use, shows four age groups. The 20-39 y r o group (orange) shows the highest volume, peaking near 500 in 2019 and 2021. The 40-59 y r o group (green) shows a steady increase to nearly 300 by 2024. The 12-19 y r o (grey) and >60 y r o (blue) groups remain below 100.

2. The second graph, Co-Use, shows similar trends but at lower volumes. The 20-39 y r o group peaks at approximately 400 in 2021. The 40-59 y r o group increases to approximately 150 by 2024. The other two age groups remain near the baseline.

Navigate back to Image 2:.

### Image 3: Long description

Panel A. The X axis represents years from 2015 to 2024. The Y axis is Percent Hospitalized ranging from 0 to 60. Four data series are shown.

* Co-Use Any Unit (solid black circles) fluctuates at the top of the graph between 45 and 55 percent.

* Single Use Any Unit (open circles) and Co-Use C C U (solid black squares) follow a similar path in the middle of the graph, starting near 25 percent, peaking in 2016, and then gradually declining toward 20 percent.

* Single Use C C U (open squares) remains the lowest series, relatively flat between 5 and 10 percent.

Panel B. The X axis represents years from 2015 to 2024. The Y axis is Percent with Serious Outcome ranging from 0 to 70. Two data series are shown.

* Co-Use (solid black circles) remains high, fluctuating between 55 and 65 percent over the decade.

* Single Use (open circles) follows a lower parallel path, fluctuating between 40 and 50 percent.

Navigate back to Image 3:.

